# Multifactor dimensionality reduction analysis identifies specific nucleotide patterns promoting genetic polymorphisms

**DOI:** 10.1186/1756-0381-2-2

**Published:** 2009-03-30

**Authors:** Eric Arehart, Scott Gleim, Bill White, John Hwa, Jason H Moore

**Affiliations:** 1 Department of Pharmacology and Toxicology, Dartmouth Medical School, Hanover, NH, USA; 2 Dartmouth-Hitchcock Medical Center, Department of Medicine (Cardiology), Lebanon, NH, USA; 3 Computational Genetics Laboratory, Department of Genetics, Norris-Cotton Cancer Center, Dartmouth Medical School, Lebanon, NH, USA; 4 Department of Biological Sciences, Dartmouth College, Hanover, NH, USA; 5 Department of Computer Science, University of New Hampshire, Durham, NH, USA; 6 Department of Computer Science, University of Vermont, Burlington, VT, USA

## Abstract

**Background:**

The fidelity of DNA replication serves as the nidus for both genetic evolution and genomic instability fostering disease. Single nucleotide polymorphisms (SNPs) constitute greater than 80% of the genetic variation between individuals. A new theory regarding DNA replication fidelity has emerged in which selectivity is governed by base-pair geometry through interactions between the selected nucleotide, the complementary strand, and the polymerase active site. We hypothesize that specific nucleotide combinations in the flanking regions of SNP fragments are associated with mutation.

**Results:**

We modeled the relationship between DNA sequence and observed polymorphisms using the novel multifactor dimensionality reduction (MDR) approach. MDR was originally developed to detect synergistic interactions between multiple SNPs that are predictive of disease susceptibility. We initially assembled data from the Broad Institute as a pilot test for the hypothesis that flanking region patterns associate with mutagenesis (n = 2194). We then confirmed and expanded our inquiry with human SNPs within coding regions and their flanking sequences collected from the National Center for Biotechnology Information (NCBI) database (n = 29967) and a control set of sequences (coding region) not associated with SNP sites randomly selected from the NCBI database (n = 29967). We discovered seven flanking region pattern associations in the Broad dataset which reached a minimum significance level of *p *≤ 0.05. Significant models (*p *<< 0.001) were detected for each SNP type examined in the larger NCBI dataset. Importantly, the flanking region models were elongated or truncated depending on the nucleotide change. Additionally, nucleotide distributions differed significantly at motif sites relative to the type of variation observed. The MDR approach effectively discerned specific sites within the flanking regions of observed SNPs and their respective identities, supporting the collective contribution of these sites to SNP genesis.

**Conclusion:**

The present study represents the first use of this computational methodology for modeling nonlinear patterns in molecular genetics. MDR was able to identify distinct nucleotide patterning around sites of mutations dependent upon the observed nucleotide change. We discovered one flanking region set that included five nucleotides clustered around a specific type of SNP site. Based on the strongly associated patterns identified in this study, it may become possible to scan genomic databases for such clustering of nucleotides in order to predict likely sites of future SNPs, and even the type of polymorphism most likely to occur.

## Background

The fidelity of DNA replication serves as the nidus for both genetic evolution and genomic instability fostering disease. Our knowledge of these processes requires an understanding of polymerase fidelity and the means by which genes are faithfully copied, proofread, and maintained in the face of environmental factors. Single nucleotide polymorphisms (SNPs) constitute greater than 80% of the genetic variation between individuals with such alterations observed every 1000 to 2000 nucleotides when comparing two human gene sequences within the genome [1]. Initially, Watson and Crick proposed that hydrogen bonding between complementary bases secured accurate DNA replication[2]. However, abundant evidence indicates the that free energy differences between correct and incorrect base pairing is not enough to account for the observed selectivity of most DNA polymerases[3]. A new theory has emerged in which selectivity is governed by base-pair geometry through interactions between the selected nucleotide, the complementary strand, and the polymerase active site[4].

Accurate DNA replication is therefore governed by correct nucleotide insertion, and in the case of polymerases with proofreading ability, by the favored extension of correctly paired complementary strands[5]. Kunkel and others proposed an "induced-fit" model for nucleotide selection where the incoming nucleotide moves the polymerase from an open to a closed configuration [3,6-8]. In this reaction mechanism, DNA polymerase binds DNA forming an "open-state" complex enabling 2'-deoxyribonucleoside 5'-triphosphate (dNTP) binding in an open ternary complex. A conformational change to the "closed-state" follows dNTP incorporation to the primer 3'-terminus. The polymerase subsequently returns to an open-state, releasing pyrophosphate (PPi) [8-10].

The overall structure of DNA polymerases is comparable to a right hand with palm, finger, and thumb domains [11]. Structural studies have shown that the templating strand bends upon exiting the polymerase catalytic site. This allows the finger domain to interact with the minor groove of the elongating strands, thereby reading the conformation of downstream base-pairing. Also, the templating strand diverts the next template base from the active site, fostering correct template reading on the part of the polymerase[12]. Not surprisingly, polymerase amino acid side-chain interactions play a critical role in efficiency and fidelity. In the case of polymerase β (pol β), Wilson and colleagues found that Asp-276 and Lys-280 form stacking interactions with the incoming nucleotide and the template, with their deletion reducing catalytic efficiency and accuracy[13]. Earlier work had shown loss of Arg-283 hydrogen bonding and van der Waals interactions with the minor groove of the templating nucleotide of the nascent base pair decreases catalysis and reduces polymerase fidelity [14-16].

Other investigators have studied the association of flanking regions on polymerase fidelity. Zhao and Boerwinkle examined neighboring-nucleotide effects on SNP genesis in the human genome and found a bias in regard to nucleotide identity in the flanking regions[17] Their work examined the proportion of each nucleotide neighboring the polymorphic site and found a large bias relative to the averages found in the human genome. This was particularly the case for positions immediately bordering the polymorphic site. Importantly, their work identified distinct bias patterns for differing transition and transversion types, as well as a bias relative to chromosome number. Subsequent work by Zhang and Zhao found neighboring-nucleotide bias in the mouse genome when compared with human SNPs[18] Our work presented here offers a more thorough picture of nucleotide bias in the flanking region of polymorphic sequences. These findings are novel in that they address synergistic interactions between nucleotide positions and the polymorphic site, adding considerable detail regarding the flanking region nucleotide patterns associated with specific transitions and transversions.

To account for the possibility of nonadditive interactions among sequences, we utilize MDR methodology developed specifically for detecting nonlinear patterns of discrete attributes predictive of a discrete endpoint. The MDR method, and associated software, was originally developed to detect interactions among genetic variations in population-based studies of disease susceptibility [19-23]. The goal of MDR is to change the representation space of the data to make nonlinear interactions easier to detect and characterize. Thus, MDR can be seen in a broader sense as a data processing step preceding classification[23]. At the heart of MDR is an attribute construction algorithm, pooling levels from multiple discrete factors to create a new discrete attribute[23].

We hypothesize that certain sequence combinations in the flanking regions of SNPs predispose toward mutation due to effects on primer strand geometry within the polymerase active site and interactions with side-chains essential for proper catalytic function, possibly altering solvation dynamics within the active site. The goal of the present study is to identify nucleotide patterns in SNP flanking regions that predispose to mutation. To accomplish this goal, we employed a novel machine learning method, multifactor dimensionality reduction (MDR), capable of identifying nonlinear patterns among discrete attributes (nucleotides) and discrete endpoints (mutation type). We found both common and unique nucleotide patterns in the flanking regions of various polymorphism types and delineated detailed associations indicative of neighboring-nucleotide effects.

## Methods

The goal of this approach is to identify combinations of nucleotides predictive of mutation type. Defining a new attribute as a function of two or more other attributes is referred to as constructive induction, or attribute construction, and was first described by Michalski et al[24]. Constructive induction using MDR is accomplished in the following way: given a threshold *T*, a combination of levels from two or more attributes, for example, is considered 'associated' with the class of interest if the ratio of class A to class B exceeds *T*; otherwise it is considered "not associated'. Once multifactor level combinations are labeled 'associated' and 'not associated' a new binary attribute is created with those two levels. Here, the classes are SNP(+) and SNP(-), with each attribute representing the nucleotide at a specific position in the flanking sequences.

**Figure 1:**
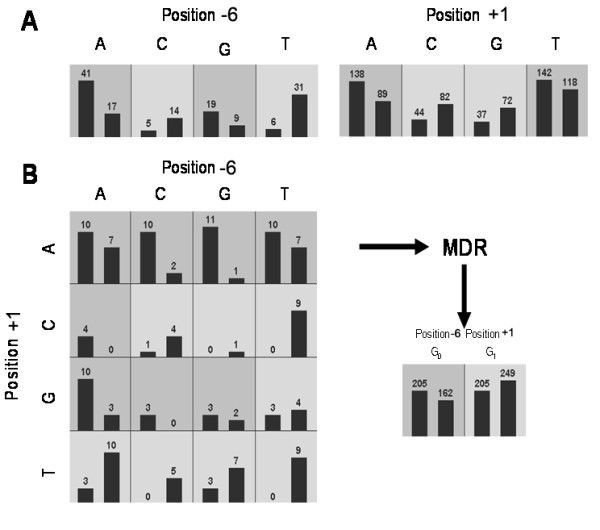
**Summary of the general steps involved in implementing the MDR method**. "High-risk" are labeled G_1 _and "low-risk" are labeled G_0 _constructing a new one dimensional attribute with G_0 _and G_1_. This list of attributes includes the flanking positions ten nucleotides long in both the 3' and 5' direction from the SNP site. Each position has four levels (A, C, G, T) or alternatively two levels (purine, pyrimidine) while the class has two levels (0, 1) that codes for controls and cases. A) illustrates distribution of cases (left bars) and controls (right bars) for each of the four possible classes of attributes. The dark shaded cells have been labeled 'high-risk' using a threshold of T = 1. The light-shaded cells have been labeled 'low-risk'. B) illustrates the distribution of cases and controls when the two functional positions are considered jointly, it is the distribution of cases and controls for the new single attribute constructed using MDR.

Once an MDR attribute is constructed it can be statistically evaluated using any classification method such as naïve Bayes, decision trees, or logistic regression[25]. It is also possible to add this new attribute back to the original dataset to be evaluated along with other attributes in a process called interleaving[25]. Computational methods such as bootstrapping, cross-validation, and permutation testing can be employed as wrappers to MDR-based constructive induction and classification to facilitate identification of a best set of predictors and their model. For the purposes of this study we change the representation of the data using MDR with the pairing of positions -6 and +1 as an example (Figure 1A) for using a naïve Bayes algorithm for classification. All possible combinations of one, two, three, and four nucleotides are evaluated for each type of SNP (Figure 1B). Training and testing accuracies are estimated using 10-fold cross-validation. The model with the highest testing accuracy is selected as the best model. Statistical significance was determined using 1000-fold permutation testing, a computationally intensive method of re-sampling datasets to empirically derive the significance of observations. The null hypothesis of no association was rejected when the upper value of the Monte Carlo *P *value derived from the permutation test was ≤ 0.05. The combination of cross-validation and permutation testing helps control the type I error rate in the presence of multiple testing. This general analytical approach has been successfully used to apply MDR in dozens of population-based association studies.

To further test our findings we employed chi square (*X*^2^) analysis to each of the datasets. In general, chi square testing demonstrated a robust level of significance (often with *p *values below the 0.0001 level) far greater than that found using the 1000-fold permutation testing approach. This is not surprising when one considers that 1000-fold permutation testing examines the predictive power of the complete flanking region model rather than each nucleotide position within that model separately.

### Broad Institute Dataset

**Figure 2:**
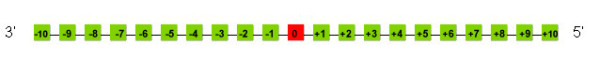
**Schematic of flanking regions in 3' and 5' direction**. Each position in the NCBI dataset was evaluated with regard to nucleotide identity, and by nucleotide and also purine/pyrimidine identity in the Broad Institute dataset.

Sequences were limited to single nucleotide polymorphisms (SNPs) from the human genome and were downloaded from The Human SNP Database (Broad Institute, MIT). SNPs sequences were collected for each of the 46 human chromosomes and classified in terms of their transition or transposition error, i.e. A/C, A/G, A/T, and so forth. Each sequence was trimmed to include 10 nucleotides in both the 3' direction (positions -10 through -1) and the 5' direction (positions +1 through +10) relative to the observed polymorphism site (at position 0 in Figure 2) so the resulting DNA polymer would include nucleotides interacting directly with the polymerase. To test the hypothesis that the location of a particular SNP carries a sequence specificity, each of these twenty attributes was assigned their nucleotide identity corresponding to the Broad Institute sequence data, i.e. adenine, cytosine, guanine, and thymine to determine which, if any, positions might confer a predisposition to replication error.

First, the SNP sequences of a given type (i.e. A or C, A or G, A or T, etc) were culled from the Broad Institute data. The 20 attributes, corresponding to 10 nucleotides in the 3' and 5' direction of the mutation site, were further classified by nucleotide type (A, G, T, or C) (four significant models are shown in Table 1). Additionally, a separate analysis was performed where nucleotide type was identified as being either a purine or a pyrimidine (three significant models are shown in Table 2). Controls were randomly selected from the general pool of sequences, not corresponding to the SNP under consideration using the MDR impute module, allowing random generation of the SNP(-) group. Each multifactoral cell in *n*-dimension space was labeled either "high association" if the SNP(+) and SNP(-) ratio met or exceeded a threshold of T = 1, and labeled "low association" if this threshold was not met. A model for each SNP(+) and SNP(-) was formed by pooling high association cells into their prospective groups. This reduced the *n*-dimension model to a one dimensional model. This was performed both on balanced and unbalanced datasets where the control group far outnumbered the study set. The MDR computational suite 6.1 allows for the use of unbalanced sets allowing greater discrimination against type II errors.

**Table 1:** Broad Dataset with Flanking Positions Identified by A, G, C, and T Character

SNP	Count	nucleotide positions	Testing accuracy.	*P-*value	*X*^2^
**A→G**	371	+1	0.558	0.05	0.001
**C→A**	84	+1	0.613	0.05	0.001
**C→G**	95	+1	0.521	<0.01	0.001
**G→A**	322	+1	0.571	0.02	0.001

MDR analysis of SNP flanking regions where upstream 3' to SNP site equals positions -10 through -1 and 5' region from the SNP site extending downstream equals position +1 through +10. All positions where evaluated with regard to nucleotide identity, i.e. adenine, cytosine, guanine, and thymine.

**Table 2:** Broad Dataset with Flanking Positions Identified as either purine or pyrimidine

SNP	Count	nucleotide positions	Testing accuracy	*P-value*	*X*^2^
**G→A**	322	+1	0.571	0.02	0.001
**T→C**	371	+2	0.536	<0.01	0.001
**T→G**	87	-2, +2	0.621	0.02	0.001

MDR analysis of SNP flanking regions where upstream 3' to SNP site equals positions -10 through -1 and 5' region from the SNP site extending downstream equals position +1 through +10. All positions were evaluated with regard to pyrimidine or purine identity.

Among all combinations of the two classes, a single model for the high risk group is constructed with the best SNP+/SNP- ratio. Single best multifactoral models are selected for each of the 2^n^-factor combinations. Then, the model with the best predictive power, having the lowest prediction error is selected. The final multifactorial model is thus selected from the classification errors and prediction errors. Statistical significance was ascertained by comparing the average cross-validation consistency of the SNP(+) sets to the value of consistencies of the SNP(-) sets (the null groups) derived from 1,000 permutations. The null hypothesis was rejected when the upper value of the Monte Carlo *P *value derived from the permutation test was = 0.05. MDR computation methods have been used previously with good success in analyzing epistatic models of disease where multiple genes interact with one another in the disease model.

### NCBI dataset

The NCBI dataset allowed us to confirm, expand, and refine the results from the Broad Institute based pilot study. We assembled 29,967 human SNP sequences into six potential nucleotide changes (Table 3). MDR analysis was used again to identify combinations of nucleotides that predict mutation type. This feature of the MDR software package allows for greater sensitivity, particularly when analyzing data with a limited number of cases. Here adjusting the threshold (*T*) and applying balanced accuracy corrects for the decreased ratio of cases to controls[26]. Statistical significance was determined using 1000-fold permutation testing.

**Table 3:** NCBI Dataset Distributions

**SNP Model**	**SNP Type**	**Occurrences**	**% of Cases**	**Exons Only**	**% exons**	**Introns Only**	**% introns**
**M**	A or C	75838	8.241639417	1981	6.591249	73857	8.297365

**R**	A or G	312882	34.00222348	11324	37.67759	301558	33.87813

**W**	A or T	58562	6.364182699	1008	3.353851	57554	6.465826

**S**	C or G	83070	9.027571749	2621	8.720679	80449	9.037934

**Y**	C or T	312689	33.98124934	11181	37.2018	301508	33.87251

**K**	G or T	76497	8.313255762	1831	6.092164	74666	8.388251

SNP model codes are based on established NCBI nomenclature. SNP type identifies the nucleotides potentially occupying the SNP site in those models.

Our NCBI data set was initially composed of 920,181 human sequences with varying SNP character. Sequences were downloaded as follows: The query, (((("homosapiens" [Organism] AND "true" [Genotype]) AND (("coding nonsynonymous" [Function class] OR "intron" [Function class]) OR "coding synonymous" [Function class])) AND "sequence" [METHODCLASS]) AND "snp" [SnpClass]), was performed on November 2, 2006 using dbSNP build 126. The resulting 920,181 records were collected in FASTA format for post-query parsing using a series of in-house developed Perl scripts. The initial records were later pruned to 29,967 due to inconsistencies in the original dataset. The first 20 nucleic acids of each sequence became an unmatched control sequence, with the requirement that control strands contain no characters other than A/a, C/c, T/t, or G/g. The 10 nucleic acids immediately flanking the identified SNP site were extracted as a case sequence. Additionally, flanking regions including 20 nucleic acids in each direction were extracted, but demonstrated no pattern association differences from the 10 nucleic acid strands. Case and control sequences were collated into tab-delimited MDR input files, with sequences labeled as case (1) or control (0), according to MDR system specifications.

## Results and discussion

It has been previously determined that certain replication errors are influenced by flanking regions adjacent to the mutation site. Small frameshifts of one-base deletions are made on undamaged DNA by DNA pol μ, pol λ, pol β, and *Escherichia coli *pol IV[27]. One such example is Streisinger slippage, resulting in simple deletions by a process of looping out of one or more bases as the primer moves along a strand of reiterated template bases[28]. This mechanism plays a role in trinucleotide expansion seen in Huntington disease, Fragile X syndrome, and Myotonic dystrophy to name a few. Other work regarding HIV Type1 reverse transcriptase (RT) found that RT side chain interactions affected polymerase fidelity and specifically that correct T-dAMP insertion was affected by the 5'-C**T**GG primer sequence in the binding pocket[5]. These studies were performed to evaluate potential independent effects of sites within the flanking regions as well as synergistic interactions between sites. Therefore, knowledge of the potential for nucleotide clusters to predispose some genomic sites to spontaneous mutation offers enormous benefit in the study of viral and bacterial mutation leading to drug resistance as well as the identification of potential pre-cancerous genetic lesions and genomic instability leading to human developmental diseases. To our knowledge, this is the first instance of MDR methods employed to evaluate the potential role of flanking regions on mutagenesis.

We tested the hypothesis that specific types of replication errors (changes to and from each combination of adenine, cytosine, guanine, or thymine) would be associated within distinct flanking region patterns. Sequences were refined from both the Broad Institute database followed by the NCBI dBSNP as described in methods. The Broad dataset, although relatively small, provides directionality of nucleotide change and would serve as an ideal pilot set to test the power of MDR. The larger NCBI set could then used to confirm, expand, and refine the identified models.

### Broad Institute Dataset

The Broad Institute dataset represented a small collection of sequences (n = 2194) compared to the larger NCBI dataset (n = 29,967) and was chosen as a pilot study to evaluate the application of MDR methodology to flanking region pattern associations with single nucleotide polymorphisms. Each position in the flanking region was identified by its specific nucleotide, generating four distinct models with positions identified as A, G, C, or T. When analyzing data sets with the nucleotide identity at each position, we discovered four SNP models that reached significance (Table 1). As we will shortly describe, four models in the Broad dataset (Table 1) were confirmed in the larger NCBI dataset. Analyzing each position as purine or pyrimidine, rather than by specific nucleotide identity, three models reached significance (Table 2). Where either G or A is observed at the SNP site, position +1 was again significantly associated with SNP occurrence (*p *= 0.02). This was consistent with results for the same dataset when positions were identified as A, G, C, or T. The Y model (C or T) showed position +2 to be significantly associated with SNP genesis of this type at the *p *< 0.01 level. And the K model (G or T) demonstrated positions -2 and +2 to be significantly associated with occurrence of the T/G polymorphism, *p *= 0.02.

To further investigate the possible contribution of flanking regions to SNP mutation sites, we classified each of the sequence positions with regard to their purine or pyrimidine identity (Table 2). This was done to explore the role of pyrimidine/purine template strand content previously found to play a role in the catalytic efficiency and fidelity of pol β and may play a role in the fidelity rate of other polymerase[13]. The same data was employed as before, with the distinction of labeling each of the ten flanking positions in the 3' and 5' direction as either purine or pyrimidine and then performing the same MDR methods as stated above.

When we examined the Broad dataset identifying the positions in the flanking regions only by their purine or pyrimidine identity we also discovered an overlapping of flanking region sets seen previously in the NCBI dataset. In this instance, the R (A or G) model again indicated significance at position +1 (*p *< 0.02). Nucleotide position +1 was also found to have significant association with the S (C or G) and K (G or T) polymorphism-type models in the Broad Institute dataset. The Y (C or T) model favors nucleotide position +2 in its motif. In the larger NCBI dataset, position +2 is included in the motif only for the Y (C or T) model, but not for the W (A or T) and K (G or T) models. Also in the Broad dataset, the K (G or T) model includes nucleotide positions -2 and +2 (*p *< 0.02), whereas in the NCBI dataset, only the Y (C or T) and R (A or G) flanking region sets include positions -2 and +2.

### NCBI Dataset

**Figure 3:**
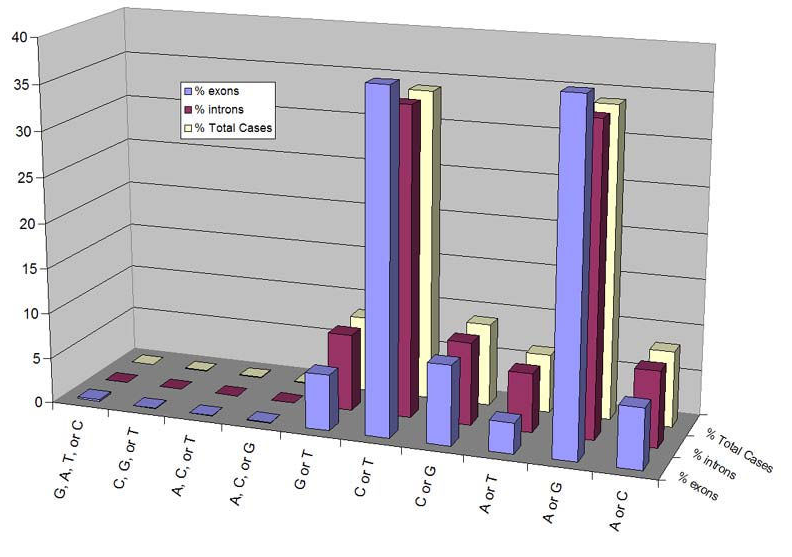
**Single Nucleotide Polymorphism dataset collected from NCBI**. Codes for cases are shown in the included table. Sequences were collected for both intronic and exonic and their numbers are given in the column marked Occurrences. Nucleotide flanking pattern searches were conducted for exonic sequences only.

**Figure 4:**
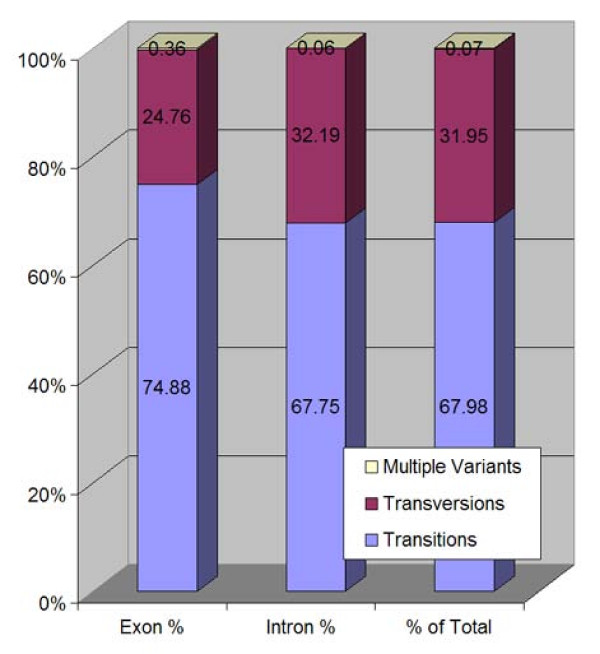
**Distribution of SNP types within the NCBI Dataset**. Representation of percent transition, transversion, and multiple variant types in both exons and introns for the NCBI dataset.

Initially we collected data for both intronic and exonic sequences (n = 909,364). The data set was then further refined to exonic sequences (n = 29,967) (Figure 3, Table 3). The dataset distribution of mutational changes was as follows: transition-type mutations (where a purine is substituted for a purine or pyrimidine for a pyrimidine) made up 74.8% of sequences identified as exonic and 67.8% of sequences identified as intronic (Figure 4, Table 4). Sequences with multiple variants were minor: 0.362% exonic, 0.0599% intronic (Figure 3, Table 4), and were excluded from the final dataset.

**Table 4:** NCBI Transition and Transversion Distributions in Exonic and Intronic Sequences

	**Exons**	**Exon %**	**Introns**	**Intron %**	**Total**	**% of Total**
**Transitions**	22505	74.87	603066	67.75	625571	67.98

**Transversions**	7441	24.75	286526	32.18	293991.8	31.94

**Multiple Variants**	109	0.362	534	0.0599	643.3627	0.06991

**Total # Records**	30055		890126		920181	

The number and percent of transition and transversion mutation types for the NCBI dataset are listed. Multiple variants are used for those SNP models that were not clearly a transition or transversion, but rather included both types of errors.

For the NCBI dataset, we found a recurring motif in the analyzed sequences for each mutation-type studied, and each motif was significant beyond the *p *<< 0.001 threshold (Table 5). Each mutation-type studied had significance at positions -1 and +1, the two positions in the templating strand directly adjacent to the mutation site (Figure 2). In the S (C or G), W (A or T), and K (G or T) SNP models the motif was expanded by one position to include positions -2, -1, and +1. The R (A or G) SNP model was further expanded by one, covering positions -2, -1, +1, and +2. The Y (C or T) SNP model demonstrated the largest motif (-3, -2, -1, +1, +2) where position -1 is adjacent to the SNP site on the 3' end and position +1 is adjacent to the SNP site on the 5' end (Table 5).

**Table 5:** NCBI Dataset Motifs

SNP Model *	**SNP Type**	**Nucleotide Positions**	**Significance**	**X**^2^
**M**	A/C	+1, +2	*P *<< 0.001	0.001
**S**	C/G	-1, +1, +2	*P *<< 0.001	0.001
**W**	A/T	-1, +1, +2	*P *<< 0.001	0.001
**K**	G/T	-1, +1, +2	*P *<< 0.001	0.001
**R**	A/G	-1, +1, +2, +3	*P *<< 0.001	0.001
**Y**	C/T	-2, -1, +1, +2, +3	*P *<< 0.001	0.001

SNP type represents identity of nucleic acid at SNP position. Motif represents positions within templating strand found to have significant association with sequences carrying an identified SNP compared to control sequences. Here position -1 is adjacent to the SNP site on the 3' end and position +1 is adjacent to the SNP site on the 5' end.

**Figure 5:**
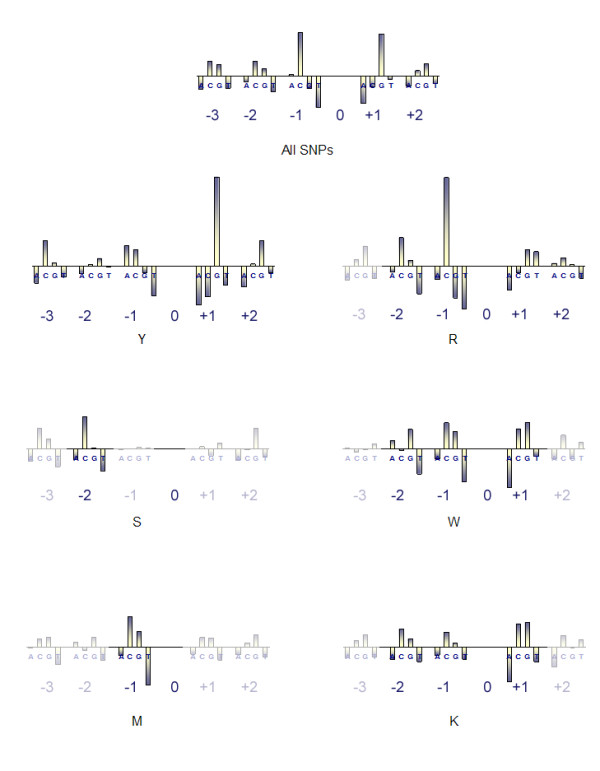
**Nucleic Acid Distribution within Each SNP Flanking Region**. The nucleotide distribution across all SNPs in our final dataset is shown center top. All four potential nucleic acids for each position within each motif are shown as bars, where a positive association is shown as a positive value on the y-axis and a negative association is depicted as a negative value on the y-axis. Nucleic acid distributions are shown for all identified SNP types.

In order to examine the contribution of nucleic acid preference within the motif, we analyzed the distribution of nucleotides at each position within the motif for each SNP type studied (Figure 5). This analysis shows a preferential distribution in each motif respective of the SNP type (Figure 5, Table 6). When examining the positions common to each SNP type motif (i.e. -1 and +1) we find that the nucleotide identity varies between SNP types. For example, in the Y SNP model position -1 is most commonly a guanine, whereas in the R, W, and K models position -1 is commonly occupied by cytosine. Interestingly, the S model distribution gives no predominant nucleic acid pattern at positions -1 and +1 (Table 6), perhaps indicating wobble to be more likely at positions -1 and +1 in the S model.

**Table 6:** Flanking nucleotide distribution for each SNP-type

SNP Model	SNP	Nucleotide Positions	Nucleotide Identity	%
Y	C/T	-3	C	**31**
		-2	G	**26**
		+1	A	**32**
		+2	G	**49**
		+3	G	**31**

R	A/G	-2	C	**32**
		-1	C	**49**
		+1	T	**30**
		+2	X	***ns***

S	C/G	-2	C	**33**
		-1	X	***ns***
		+1	X	***Ns***

W	A/T	-2	G	**29**
		-1	C	**31**
		+1	G	**31**

K	G/T	-2	C	**29**
		-1	C	**28**
		+1	G	**31**

SNP models are identified by their respective NCBI single letter code and by their potential mutation identity. Nucleotide frequency is given as percent, with a lack of significance denoted as *ns*.

In addition to permutation testing for these models we also performed *X*^2 ^as an alternative method. All models demonstrated significance below or at the *p *= 0.001 level. This is not surprising given *X*^2 ^tendency to over predict synergistic models [24]. Ultimately, MDR takes a more conservative approach to significance testing due to the requirement that the models are tested as a unit rather than as individual contributions to the model.

## Conclusion

Our analysis of two datasets has shown the existence of neighboring nucleotide patterns that persist across identified single-nucleotide polymorphism (SNP) models. Importantly, this pattern grows to include additional nucleotide positions depending on the type of polymorphism observed. We have also found that for a given SNP type, the distribution of nucleotides within the flanking region shows specificity in association with certain SNP types. Comparison of the Broad Institute dataset with the NCBI dataset demonstrated an overlap in flanking region sets and provided some directionality with regard to SNP genesis. Such studies will allow for the development of more powerful and predictive algorithms offering the possibility of predicting both occurrence and direction of SNP genesis *in vivo*.

## Competing interests

The authors declare that they have no competing interests.

## Authors' contributions

EA and SG devised the experimental approach and drafted the manuscript. SG collected the genomic sequences, calculated nucleotide distributions at each site, and parsed the polymorphic sequences into MDR input files. EA and BW carried out the MDR calculations, permutation testing, and Chi-square analysis. JH and JM participated in the design, coordination, and interpretation of the study. All authors read and approved the final manuscript.
